# CAR T-cell therapy induced pseudoprogression

**DOI:** 10.3389/fimmu.2026.1691562

**Published:** 2026-03-19

**Authors:** Erin A. Dean

**Affiliations:** Hematopoietic Stem Cell Transplantation and Cellular Therapy Program, Division of Hematology/Oncology, Department of Medicine, University of California Irvine, Orange, CA, United States

**Keywords:** CAR T-cell therapy, CARTiPP, checkpoint inhibitors, hematologic malignancies, PET/CT scan, pseudoprogression, solid tumors, tumor microenvironment

## Abstract

Pseudoprogression is defined as a seeming post-therapy increase in tumor burden on imaging that recedes on subsequent reimaging without additional therapy. The origins of pseudoprogression lie in the field of immunology with current understanding of the imaging finding coming mostly from experience with checkpoint inhibitors used for management of solid tumors. With the wider administration of chimeric antigen receptor (CAR) T-cell therapy for the treatment of hematologic malignancies, CAR T-cell induced pseudoprogression (CARTiPP) has also been noted although it has remained an underreported, although likely rare, adverse event lacking clear description and management guidelines. CARTiPP can obscure response assessment in patients and lead to serious clinical complications due to edema-driven compression of organs or tissue. This mini review summarizes the emergence of pseudoprogression with the rise of immunotherapy along with the modifications of radiologic guidelines for correct interpretation of scans as a key non-invasive technique for overcoming the associated diagnostic challenges. It discusses the limited available data on the nature of CARTiPP in the setting of the tumor microenvironment and its meaning for the care of patients receiving CAR T-cell therapy.

## Introduction

Chimeric antigen receptor (CAR) T-cell therapy has picked up speed for the treatment of hematologic malignancies and most recently solid tumors and autoimmune disorders (1). Currently, the commercially available products include anti-CD19 axicabtagene ciloleucel (axi-cel), lisocabtagene maraleucel, tisagenlecleucel, brexucabtagene autoleucel, and obecabtagene autoleucel for relapsed/refractory (R/R) lymphomas and/or B-cell precursor acute lymphoblastic leukemia (2, 3); anti-B-cell maturation antigen (BCMA) idecabtagene vicleucel and ciltacabtagene autoleucel for R/R multiple myeloma (4); and anti-melanoma-associated antigen A4 afamitresgene autoleucel for unresectable/metastatic synovial sarcoma (5). After a single treatment, best response can occur over time, typically in weeks to months (6). To definitively determine response to therapy, obtaining a clear radiology read of studies is crucial. Interpretation of scans needs to be uniform and fully reliable. However, a phenomenon known as pseudoprogression noted on imaging can obscure the direction of response, and thus, the necessary next steps in clinical care. The purpose of this work is to explain pseudoprogression in the context of immunotherapy and provide a summary of the latest information as it pertains to CAR T-cell therapy.

## Background

Pseudoprogression is defined as a transient apparent increase in tumor burden, either due to enlarging existing lesion(s) or the appearance of new lesion(s), on imaging (7). In contrast to true disease progression, in pseudoprogression, the next reimaging study demonstrates a decrease in tumor burden, without change in treatment line (7). Tumor flare is a related phenomenon which causes clinical symptoms, including pain or fever, with or without increase of tumor size on imaging (8).

The term pseudoprogression was first coined in 2009 in a case series describing the radiologic images of 3 patients with metastatic melanoma who received the immunotherapy drug ipilimumab (9). Immune checkpoint inhibitors, including programmed death-ligand 1 (PD-L1), programmed death-1 (PD-1), and cytotoxic T-lymphocyte antigen-4 (CTLA-4) used in the management of variety of solid tumors, constitute the most common type of cancer treatment associated with pseudoprogression with rates varying by type of tumor and drug, generally ranging from 2 to 10% (10, 11). There has been a higher incidence of pseudoprogression observed in young patients, which has been potentially attributed to a more robust immune response to therapy (12). In general, however, pseudoprogression is associated with shorter duration of response (11). The timing of pseudoprogression in the setting of immune checkpoint inhibitors has been well described with the finding being labeled as early versus delayed depending on if it occurs before or after 12 weeks of treatment initiation, respectively (7).

Since immune checkpoint inhibitors work by immune and T cell activation (11), the extent and speed of immune cell infiltration of the tumor can also lead to necrosis, hemorrhage, or edema of the surrounding tissues (7), and affect the time to response. A few theories have been proposed to explain the occurrence of pseudoprogression, namely infiltration of the tumor by T cells (13), increased inflammation in the tumor due to the release of cytokines (9), and true tumor progression before the adaptive immune response is mounted (14).

Work up for pseudoprogression involves assessing for other entities, such as infection due to patients’ immunocompromised status, hyperprogression, and sarcoidosis-like reactions. Hyperprogression, which is associated with PD-L1 and PD-1 therapies, presents with paradoxical, persistent, rapid tumor growth ultimately leading to lack of response to therapy (11). Sarcoidosis-like reactions, which are associated with CTLA-4 drug use, are persistent granulomatous formations that can be distinguished from cancer on pathology (15).

## Updated radiologic guidelines and radiomics

### Modified response evaluation criteria in solid tumors

The advent of new types of immunotherapies, including checkpoint inhibitors, cancer vaccines, oncolytic virus, and adoptive cell transfer, has necessitated modification of the response evaluation criteria in solid tumors (RECIST) 1.1, which at present serve as the gold standard for radiologic assessment of treatment response in solid tumors on either chest X-ray, computed tomography (CT), magnetic resonance imaging (MRI), or 18F-fluorodeoxyglucose (^18^F-FDG) positron emission tomography (PET) (16). To allow improved image interpretation in the setting of immunotherapy, modified criteria have been formulated. The immune-related response criteria (irRC) were released in 2009, followed by immune-related RECIST (irRECIST) in 2013, immunotherapy response assessment in neuro-oncology (iRANO) in 2015, immune RECIST (iRECIST) in 2017, and immune-modified RECIST (imRECIST) in 2018 (16–18). Given the different patterns of response observed with immunotherapies, the goal of the modified criteria has been to go beyond assessing only morphologic change of tumor size but also tumor metabolic activity, viability, and attenuation (16). Modified criteria have incorporated two new concepts: (1) measuring and adding new lesions into disease assessments and (2) performing a repeat scan at least 4 weeks from prior to confirm progressive disease (PD) (11). **Table 1** lists the standard and updated radiologic guidelines and compares each set’s definition of PD (11, 16–20). It should be noted that since they rely mostly on data from melanoma and non-small cell lung cancer, these guidelines may lack generalizability (11). Due to convenience of use, irRECIST and imRECIST have been preferentially applied in clinical trials (18).

**Table 1:** Main modifications in response assessment criteria in solid tumors and lymphoma following treatment with immunomodulatory therapy.

Criteria	Year	Definition of PD	
Solid Tumors		
RECIST 1.1	2009	≥ 20% ↑ in sum of longest diameters with ↑ ≥ 5 mm, or new lesions ​ lesion: 10 mm on CT/MRI (slice thickness ≤ 5 mm), or 2 x slice thickness (slice thickness > 5 mm); on PET, included only in the detection of new lesions lymph node: ≥ 15 mm is measurable, target lesion; 10–14 mm is non-measurable, non-target lesion maximum 5 lesions (≤ 2 per organ)
irRC	2009	≥ 25% ↑ in sum of target and new lesions ​ 5 lesions (≥ 5 x 5 mm) per organ (up to 10 visceral and 5 cutaneous)
irRECIST	2013	≥ 20% ↑ in sum of target and new lesions ​ definitions of target lesion, non-target lesion, lymph node same as RESIST 1.1: maximum 5 lesions (2 per organ) (≥ 10 mm in diameter; ≥ 15 mm for nodal lesions)
iRANO	2015	≥ 25% ↑ in enhancing lesions; ↑ in non-enhancing FLAIR/T2W lesions; new lesions ​ measurable lesion: two perpendicular diameters of at least 10 mm, and visible on 2 axial slices which are preferably at least 5 mm apart
iRECIST	2017	≥ 5 mm ↑ in tumor burden or new lesions ​ definitions of target lesion, non-target lesion, lymph node same as RESIST 1.1: maximum 5 lesions (2 per organ) (≥ 10 mm in diameter; ≥ 15 mm for nodal lesions)
imRECIST	2018	≥ 20% ↑ in sum of target and new lesions; new lesions not PD ​ definitions of target lesion, non-target lesion, lymph node same as RESIST 1.1: maximum 5 lesions (2 per organ) (≥ 10 mm in diameter; ≥ 15 mm for nodal lesions)
Lymphoma		
Lugano	2014	PET-CT score 4 or 5 ​ Regrowth of previously resolved lesions New node > 15 mm or new extranodal site > 10 mm in any axis; if smaller size lesion, must be unequivocally attributable to lymphoma
LYRIC	2016	Definitions of lesions same as Lugano plus: IR (1): ≥ 50% ↑ SPD in 12 weeks IR (2): < 50% ↑ SPD + new lesion IR (3): FDG ↑ ​

PD, progressive disease; RECIST, response evaluation criteria in solid tumors; irRC, immune-related response criteria; irRECIST, immune-related RECIST; iRANO, immunotherapy response assessment in neuro-oncology; iRECIST, immune RECIST; imRECIST, immune-modified RECIST; LYRIC, lymphoma response to immunomodulatory therapy criteria; PET/CT, positron emission tomography/computed tomography; IR, indeterminate response; SPD, sum of the products of the diameters; ↑, increase.

### Lymphoma response to immunomodulatory therapy criteria

Likewise, with the release of new lymphoma immunotherapies, the lymphoma response to immunomodulatory therapy criteria (LYRIC) were created to flag possible pseudoprogression versus PD on a scan as indeterminate response (IR) that requires a repeat assessment in 12 weeks for a final determination (21). There are 3 types of IR lesions, 1-3, each with a definition of what constitutes PD: type 1, ≥ 50% increase in sum of the products of the diameters (SPD) of up to 6 measurable lesions; type 2, ≤ 50% increase in SPD of up to 6 measurable lesions plus a new lesion; and type 3, increase in FDG (21). LYRIC were an update to the Lugano criteria, which were originally developed to interpret ^18^F-FDG-PET scans of patients with lymphoma following receipt of chemotherapy or chemoimmunotherapy, incorporating rituximab (21) (**Table 1**).

### Metabolic tumor volume

Determination of total metabolic tumor volume (MTV), which captures all active tumor in the body on PET/CT, has also been assessed as an imaging tool to distinguish pseudoprogression from true progression or physiologic background. In one study, the change in MTV from baseline to first assessment in patients with R/R Hodgkin lymphoma receiving PD-1 blockade therapy (including either nivolumab or pembrolizumab) predicted treatment response and outcomes, particularly in those with initial IR (22). In another study, in patients with metastatic non-small cell lung cancer treated with immune checkpoint inhibitors, MTV on interim PET/CT 6–8 weeks after the start of treatment complemented the PET response criteria in solid tumors (PERCIST) with higher MTV supporting the continuation of treatment in patients deemed to be progressing per PERCIST (23). MTV is considered a more reliable measurement compared to another potential prognostic radiologic marker, total lesion glycolysis (TLG), which is obtained by multiplying MTV by the mean standardized uptake value (SUV_mean_) for each hypermetabolic lesion, since SUV_mean_ depends on the imaging technique (23). Although maximum SUV, which captures the uptake of the highest voxel in the most intense lesion on PET/CT, can also have prognostic potential (23), its measurement can be affected by image noise (22). The advantage of MTV is that it is a potential comprehensive, non-invasive method for pseudoprogression evaluation that can be derived off a standard PET/CT scan, although its reproducibility and speed of derivation are still being developed for use in practice.

### CAR T-cell induced pseudoprogression

CAR T-cell therapy was first approved by the Food and Drug Administration for commercial use in 2017 (1). The therapy works by targeting an antigen on the malignant cell (1), triggering a local and a wider systemic immune response aimed at eliminating the tumor. Compared to the more common adverse events cytokine release syndrome (CRS) and immune effector cell-associated neurotoxicity syndrome (ICANS), CAR T-cell induced pseudoprogression (CARTiPP) is a rare and less known complication of CAR T-cell therapy that lacks available complete data on prevalence, clear characterization, or management strategies (24).

### Prevalence and complications

A handful of larger studies have reported on the incidence of CARTiPP. In one retrospective study, 10% of patients (n= 8) who received either anti-CD19 or anti-BCMA CAR T-cell therapy over a 46-month period developed CARTiPP within 10 days of treatment, with 6 experiencing clinical complications ≥ Grade 2 Common Terminology Criteria of Adverse Events; 7 had CRS and 3 ICANS (24). In a group of patients with secondary central nervous system non-Hodgkin lymphoma receiving anti-CD19 CAR T-cell therapy, 7 out of 26 patients (27%) were reported to have pseudoprogression on MRI Brain (25). In another study, 6 out of 115 patients (5.2%) with non-Hodgkin lymphoma receiving anti-CD19 CAR T-cell therapy displayed pseudoprogression at median of +6.5 (range 2-21) days; all had CRS and 2 ICANS (26). In a phase 1b/2 in house anti-CD19 CAR-cell therapy study, 3 out of 56 patients (5%) were found to have clinical and radiological signs of CARTiPP at +7–10 days post therapy; all had concurrent CRS and 2 ICANS (27). Finally, in a radiomics study, 3 out of 19 patients (16%) who received anti-CD19 CAR T-cell therapy were suspected to have CARTiPP at + 4–5 days post infusion, with a higher MTV in those with clinical symptoms; 1 patient had CRS and none ICANS (28).

Additionally, a number of individual cases of CARTiPP have been recorded with events presenting either early (< +10 days) (29, 30) or delayed (> +10 days) (31), (at +3 and +9 months) (32) post therapy. Some events caused end-organ damage, such as ocular complications (33–35). A patient with a solid tumor, gastric cancer, treated on a clinical trial of Claudin 18.2 CAR T-cell therapy was also noted to have CARTiPP at +1 month (36).

In addition to apparent progression of disease on imaging, pseudoprogression in the setting of CAR T-cell therapy can occasionally lead to serious clinical complications. Especially when it happens early post infusion, it can cause compression effects on vital organs due to tumor swelling, which is why bridging therapy may be considered for preemptive tumor burden reduction and corticosteroids, tocilizumab or cyclophosphamide may be used for active management (37).

Overall, there are more documented cases of early than delayed CARTiPP. In one study where biopsies were obtained from 11 patients (10 with PD, 1 with partial response), all were found to have tumor rather than inflammation, possibly because the median time to a first scan was 86 days and CARTiPP is mostly an acute event (38).

### Tumor microenvironment

Biopsying lesions that appear to progress on imaging may provide a definitive diagnosis, but it is not always feasible.

Dissecting the tumor microenvironment (TME), where the CAR T-cells act in concert with the immune system against the malignancy, is central in understanding pseudoprogression. The TME consists of tumor cells, drafted immune cells, new blood vessels and lymphatics, and milieu of inflammatory proteins (39). The immune response in general is executed through a series of actions including dendritic cell presentation of antigens, released from killed tumor cells, via the major histocompatibility complex (a process bypassed in CAR T-cell therapy); T cell activation by cytokines and T cell recruitment by chemokines; and killing of tumor cells by activated T cells via various secreted enzymes (39).

The breakdown in these anti-tumor mechanisms, coupled with the development of immunosuppression in the TME, leads to tumor growth (39). Immunosuppressive conditions are created through epigenetic aberrations, DNA methylation, differentiation of T cells into regulatory T (Treg) cells, and alterations in levels of reactive oxygen species, glucose, and amino acids in the TME (39).

Multiple factors in the TME have been identified as interfering with the anti-tumor effects of CAR T-cell therapy. Certain chemokines and cytokines can pull in immunosuppressive cells including Treg cells, tumor-associated macrophages (TAMs), and myeloid-derived suppressor cells (MDSCs) (40). To eliminate cytotoxic T cells functioning, Tregs act by releasing cytokines, consuming intrleukin-2, suppressing antigen-presenting cells, and halting T cell activation; TAMs secrete cytokines, amino-acid-depleting enzymes, and recruit more Tregs; while MDSCs directly aim at effector T cells (40). The environment in the TME becomes more immunosuppressive also via metabolites like indoleamine 2,3- dioxygenase, which increases the number of and actives MDSCs and suppresses effector T cells and natural killer cells in the TME; and lactate, which reduces T cell signaling and increases Tregs and M2 macrophages (40). Additionally, tumor cells secrete immunosuppressive extracellular vesicles, which can render CAR T-cells dysfunctional (41). The relationship between tumor cells, CAR T-cells, and the TME is fluid with immunity being polarized either towards an anti-tumor or an immunosuppressive direction depending on various factors.

The immune state of the TME helps determine the patient’s initial response to cellular immunotherapy. In one study of the TME, pre-CAR T-cell therapy and +2 week-tumor samples from 51 patients with R/R large B-cell lymphoma (LBCL) who received axi-cel were systematically investigated and associated with clinical response on imaging (42). The TME underwent transformative changes during the 2-week period and differed between responders and non-responders (42). In responders, the gene expression in both the adaptive and innate immune system increased with changes in T cell cytotoxicity, T cell growth factor, interferon-γ–regulated immune checkpoint, myeloid, and chemokines related genes (42). Also, in responders, the gene expression for B cell lineage markers decreased including CD19, CD20, CD22, PAX5, ST6GAL1, POU2AF1, and cancer testis antigens (42). The TME of patients with favorable clinical outcomes was consistent with anti-tumor inflammation, including T cell infiltration, chemokines, CCL5 and CCL22, γ-chain receptor cytokines, IL-15, IL-7 and IL-21, and interferon-regulated molecules; versus in those with unfavorable clinical outcomes which was consistent with immunosuppressive tumor growth, including pro-inflammatory chemokines, CXCL10 and CXCL11 (42). Examination of these tissue biopsies highlighted the rapid inflammatory response occurring within the TME post CAR T-cell therapy. In a similar study of patients treated with axi-cel for LBCL, 18 post-treatment biopsies were analyzed that found increase in pan-macrophages, cytotoxic T cells [CD8 naïve T cells; activated cytotoxic T cells (GZMB+); PD1+TIM3+LAG3- cytotoxic T cells; CD8 regulatory T cells (FOXP3+)], T helper cells (CD4 naive T cells, T helper Th2, cytotoxic T lymphocyte TC2, and TC1/TC2 ratio) in durable response cases; and spatial concentration of M-MDSC in relapsed cases (43). This tug of war between a pro-inflammatory, anti-tumor and immunosuppressive, pro-tumor response that occurs acutely likely represents the reason behind radiologically and/or clinically observed pseudoprogression.

In the future, conceptualization of the TME may improve through newer technologies such as multiplex tissue imaging and spatial analysis (44). In contrast to performing standard immunohistochemistry on a formalin-fixed paraffin-embedded block of tissue, multiplex imaging can detect dozens of different antigens at once thus leading to identification of each cell type (for example, tumor versus immune cells) (44). Completion of cell phenotyping then allows spatial analysis of the cells in terms of distribution of cell types, composition of repeated cell populations, structure within the tissue, distance between cells, and communication between cells modeled by mathematics (44). The benefit of mapping the arrangement of cells within the TME is deriving their functional status as it can depend on their 3D location (44). More easily examining tissues over time facilitates tracking changes in the role of some cells that can perform either anti- or pro- tumor tasks (42). These new diagnostic technologies are close to being used in clinical practice and will speed up cell and marker identification to allow possible modulation of pathways and improvement in CAR T-cell therapy.

### Non-invasive evaluation of CARTiPP

Differentiating CARTiPP from PD on imaging can be challenging especially without other signs or symptoms of worsening disease. In addition to applying the updated radiologic guidelines, determining MTV and TLG, which serve as quantitative measures of active tumor on PET/CT, may be used to understand the status of the tumor post CAR T-cell therapy (28).

Circulating tumor (ct) DNA can be employed as a supplemental peripheral test to scans (45). In R/R LBCL, pre-lymphodepletion ctDNA and MTV have been shown to correlate (44), and day +28 ctDNA has been found to be prognostic for patients in partial remission or stable disease on simultaneous PET/CT assessment with detectable serum levels associated with future progression of disease (46, 47). On day +28, lack of correlation between serum ctDNA and MTV, however, has been attributed to ongoing inflammation at the site of the tumor (48). Finalization of the response to therapy with resolution of inflammation, which if not reached by day +28 typically occurs by the next standard assessment in 2 months, is hypothesized to be the reason behind the positive correlation between the two markers at that later time point on day +90 (48). While ctDNA has been checked on day +7, day +14, day +21 in CAR T-cell therapy recipients (46), it has not yet been assessed in those experiencing CARTiPP or correlated with MTV at the same time points to elucidate dynamics of tumor burden. Since CARTiPP typically occurs early on post-CAR T-cell therapy infusion, this will be necessary for ctDNA and MTV to be developed as biomarkers for CARTiPP monitoring.

Additionally, elevation in interleukin-8 (IL-8) in the blood has been proposed as another non-invasive diagnostic biomarker of CARTiPP (30). IL-8 is a CXC chemokine for neutrophils that is secreted by different solid and hematologic malignant cells and tumor stromal cells with levels corresponding to tumor burden (49). Decreasing IL-8 levels compared to pre-treatment have been shown to indicate pseudoprogression in patients with non-small cell lung cancer and bladder cancer receiving checkpoint inhibitors (50). Serum IL-8 levels from frozen samples retrospectively tested weekly post anti-CD19 CAR T-cell therapy for aggressive non-Hodgkin lymphoma in patients with CARTiPP remained low in 2 patients and peaked day +7 and decreased day +14 in 1 patient (49). Determining if CARTiPP is associated with CRS or ICANS, which also predominantly occur acutely, and if other inflammatory markers can be used to predict pseudoprogression requires further investigation.

## Conclusion

While pseudoprogression due to checkpoint inhibitors has been well described in the literature, CARTiPP remains an unexplored area of study within the field of immunotherapy. This is most likely due to the fact that CARTiPP tends to be an acute event that typically occurs shortly after CAR T-cell therapy infusion and prior to the first standard PET/CT assessment at +1 month, and early imaging and biopsies are infrequently obtained for investigation unless clinically necessary. Given its timing, concurrent CRS and ICANS are common. Since CARTiPP is less frequently observed as a delayed event at the +1 month and beyond post-infusion timepoints, true progression of disease is favored upon increase of metabolic activity on those restaging PET/CT scans. Even though a tissue biopsy can directly characterize the etiology of radiologically worsened lesions, supplementary non-invasive imaging and laboratory methods are also being developed to aid diagnosis. Awareness of CARTiPP is important for accurate response determination and appropriate adverse event management. Prospective studies as well as consistent reporting of CAR T-cell therapy related toxicities can lead to better understanding of the mechanism of action of CAR T-cell therapy within the TME that causes CARTiPP and can help create guidelines for both monitoring and providing clinical care to patients with CARTiPP.

## References

[1] Mitra A Barua A Huang L Ganguly S Feng Q He B . From bench to bedside: the history and progress of CAR T cell therapy. Front Immunol. (2023) 14:1188049. doi: 10.3389/fimmu.2023.1188049, PMID: 37256141 PMC10225594

[2] B-cell lymphomas. Version 2.2026 – June 24, 2025. National Comprehensive Cancer Network Clinical Practice Guidelines in Oncology. NCCN.org. 10.6004/jnccn.2022.0015 35276674

[3] Acute lymphoblastic leukemia. CAR T cells. Version 2.2025 – June 27, 2025. National Comprehensive Cancer Network Clinical Practice Guidelines in Oncology. NCCN.org. 10.6004/jnccn.2025.0006 39938467

[4] Multiple myeloma. CAR T-cell therapies. Version 5.2026 – January 9, 2026. National Comprehensive Cancer Network Clinical Practice Guidelines in Oncology. NCCN.org. 10.6004/jnccn.2026.0001 41671464

[5] D’Angelo SP Araujo DM Abdul Razak AR Agulnik M Attia S Blay JY . Afamitresgene autoleucel for advanced synovial sarcoma and myxoid round cell liposarcoma (SPEARHEAD-1): an international, open-label, phase 2 trial. Lancet. (2024) 403:1460–71. doi: 10.1016/S0140-6736(24)00319-2, PMID: 38554725 PMC11419333

[6] Wittibschlager V Bacher U Seipel K Porret N Wiedemann G Haslebacher C . CAR T-cell persistence correlates with improved outcome in patients with B-cell lymphoma. Int J Mol Sci. (2023) 24:5688. doi: 10.3390/ijms24065688, PMID: 36982764 PMC10056741

[7] Bedmutha A Kaushal A . Pseudoprogression during immunotherapy in metastatic head and neck squamous cell carcinoma. Radiol Imaging Can. (2022) 4:e220037. doi: 10.1148/rycan.220037, PMID: 35549358 PMC9152687

[8] Taleb BA . Tumour flare reaction in cancer treatments: a comprehensive literature review. Anticancer Drugs. (2019) 30:953–8. doi: 10.1097/CAD.0000000000000814, PMID: 31348010 PMC6749970

[9] Di Giacomo AM Danielli R Guidoboni M Calabrò L Carlucci D Miracco C . Therapeutic efficacy of ipilimumab, an anti-CTLA-4 monoclonal antibody, in patients with metastatic melanoma unresponsive to prior systemic treatments: clinical and immunological evidence from three patient cases. Cancer Immunol Immunother. (2009) 58:1297–306. doi: 10.1007/s00262-008-0642-y, PMID: 19139884 PMC11030873

[10] Soria F Beleni AI D’Andrea D Resch I Gust KM Gontero P . Pseudoprogression and hyperprogression during immune checkpoint inhibitor therapy for urothelial and kidney cancer. World J Urol. (2018) 36:1703–9. doi: 10.1007/s00345-018-2264-0, PMID: 29549485 PMC6208670

[11] Dromain C Beigelman C Pozzessere C Duran R Digklia A . Imaging of tumour response to immunotherapy. Eur Radiol Exp. (2020) 4:2. doi: 10.1186/s41747-019-0134-1, PMID: 31900689 PMC6942076

[12] Nishino M Giobbie-Hurder A Manos MP Bailey N Buchbinder EI Ott PA . Immune-related tumor response dynamics in melanoma patients treated with pembrolizumab: identifying markers for clinical outcome and treatment decisions. Clin Cancer Res. (2017) 23:4671–9. doi: 10.1158/1078-0432.CCR-17-0114, PMID: 28592629 PMC5559305

[13] Wolchok JD Hoos A O’Day S Weber JS Hamid O Lebbé C . Guidelines for the evaluation of immune therapy activity in solid tumors: immune-related response criteria. Clin Cancer Res. (2009) 15:7412–20. doi: 10.1158/1078-0432.CCR-09-1624, PMID: 19934295

[14] Gainor JF Longo DL Chabner BA . Pharmacodynamic biomarkers: falling short of the mark? Clin Cancer Res. (2014) 20:2587–94. doi: 10.1158/1078-0432.CCR-13-3132, PMID: 24831281

[15] Pozzessere C Mazini B Omoumi P Jreige M Noirez L Digklia A . Immune-related adverse events induced by immune checkpoint inhibitors and CAR-T cell therapy: A comprehensive imaging-based review. Cancers (Basel). (2024) 16:2585. doi: 10.3390/cancers16142585, PMID: 39061225 PMC11274393

[16] Ko CC Yeh LR Kuo YT Chen JH . Imaging biomarkers for evaluating tumor response: RECIST and beyond. biomark Res. (2021) 9:52. doi: 10.1186/s40364-021-00306-8, PMID: 34215324 PMC8252278

[17] Okada H Weller M Huang R Finocchiaro G Gilbert MR Wick W . Immunotherapy response assessment in neuro-oncology: a report of the RANO working group. Lancet Oncol. (2015) 16:e534–42. doi: 10.1016/S1470-2045(15)00088-1, PMID: 26545842 PMC4638131

[18] Kataoka Y Hirano K . Which criteria should we use to evaluate the efficacy of immune-checkpoint inhibitors? Ann Transl Med. (2018) 6:222. doi: 10.21037/atm.2018.04.17, PMID: 30023385 PMC6035987

[19] Ellingson BM Sampson J Achrol AS Aghi MK Bankiewicz K Wang C . and standard RANO response to convection-enhanced delivery of IL4R-targeted immunotoxin MDNA55 in recurrent glioblastoma. Clin Cancer Res. (2021) 27:3916–25. doi: 10.1158/1078-0432.CCR-21-0446, PMID: 33863808 PMC8282697

[20] Ferrari C Maggialetti N Masi T Nappi AG Santo G Niccoli Asabella A . Early evaluation of immunotherapy response in lymphoma patients by 18F-FDG PET/CT: A literature overview. J Pers Med. (2021) 11:217. doi: 10.3390/jpm11030217, PMID: 33803667 PMC8002936

[21] Cheson BD Ansell S Schwartz L Gordon LI Advani R Jacene HA . Refinement of the Lugano Classification lymphoma response criteria in the era of immunomodulatory therapy. Blood. (2016) 128:2489–96. doi: 10.1182/blood-2016-05-718528, PMID: 27574190

[22] Wills B Michaud L Schöder H Ganesan N Moskowitz CH Straus DJ . Quantitative change in metabolic tumor volume may assist in distinguishing between pseudoprogressors and responders in patients with relapsed/refractory classical hodgkin lymphoma treated with PD-1 blockade. Blood. (2021) 138. doi: 10.1182/blood-2021-153383, PMID: 41761659

[23] Tricarico P Chardin D Martin N Contu S Hugonnet F Otto J . Total metabolic tumor volume on ^18^F-FDG PET/CT is a game-changer for patients with metastatic lung cancer treated with immunotherapy. J Immunother Can. (2024) 12:e007628. doi: 10.1136/jitc-2023-007628, PMID: 38649279 PMC11043703

[24] Schroeder T Martens T Chitadze G Yalcin F Trautmann H Brüggemann M . Pseudoprogression as adverse event of chimeric antigen receptor T cell therapy. Blood. (2023) 142:6935. doi: 10.1182/blood-2023-189047, PMID: 41761659

[25] Hernández-Tost H Weiss N Choquet S Birzu C Le Guennec L Mersali S . Neurotoxicity in patients with CNS lymphomas treated with CAR T-cell therapy: A study from the french oculo-cerebral lymphoma network. Neurology. (2025) 104:e213501. doi: 10.1212/WNL.0000000000213501, PMID: 40146949

[26] Sortais C Cordeil S Bourbon E Idlhaj M Ferrant E Safar V . Flare-up phenomenon or pseudoprogression after CAR T-cell infusion in non-Hodgkin aggressive lymphomas. Leuk Lymphoma. (2023) 64:707–11. doi: 10.1080/10428194.2022.2161304, PMID: 36573418

[27] Danylesko I Shouval R Shem-Tov N Yerushalmi R Jacoby E Besser MJ . Immune imitation of tumor progression after anti-CD19 chimeric antigen receptor T cells treatment in aggressive B-cell lymphoma. Bone Marrow Transplant. (2021) 56:1134–43. doi: 10.1038/s41409-020-01156-y, PMID: 33268830 PMC8113054

[28] Wang J Hu Y Yang S Wei G Zhao X Wu W . Role of fluorodeoxyglucose positron emission tomography/computed tomography in predicting the adverse effects of chimeric antigen receptor T cell therapy in patients with non-hodgkin lymphoma. Biol Blood Marrow Transplant. (2019) 25:1092–8. doi: 10.1016/j.bbmt.2019.02.008, PMID: 30769193

[29] Boursier C Perrin M Bordonne M Campidelli A Verger A . Early 18F-FDG PET flare-up phenomenon after CAR T-cell therapy in lymphoma. Clin Nucl Med. (2022) 47:e152–3. doi: 10.1097/RLU.0000000000003870, PMID: 34406181

[30] Zhong N Ma Q Gong S Shi Y Zhao L Wang D . Rapid response in relapsed follicular lymphoma to novel anti-CD19 CAR-T therapy with pseudo-progression and cytomegalovirus infection: A case report. Int Immunopharmacol. (2024) 134:112174. doi: 10.1016/j.intimp.2024.112174, PMID: 38703571

[31] Fukushima K Ueda T Takamori H Isohashi K Matsunaga K Tanaka Y . PET-CT positive lesions suggestive of pseudoprogression during long-term follow-up after complete remission with CAR-T cell therapy. Rinsho Ketsueki. (2024) 65:1363–7. doi: 10.11406/rinketsu.65.1363, PMID: 39935222

[32] Cutini I . La pseudoprogressione post CAR-T: amico o nemico? [Pseudoprogression after CAR-T cell therapy: friend or foe]? Recenti Prog Med. (2025) 116:4e–7e. doi: 10.1701/4416.44125, PMID: 39840879

[33] Karschnia P Rejeski K Winkelmann M Schöberl F Bücklein VL Blumenberg V . Toxicities and response rates of secondary CNS lymphoma after adoptive immunotherapy with CD19-directed chimeric antigen receptor T cells. Neurology. (2022) 98:884–9. doi: 10.1212/WNL.0000000000200608, PMID: 35351785 PMC9169944

[34] Denton CC Gange WS Abdel-Azim H Jodele S Kapoor N Oberley MJ . Bilateral retinal detachment after chimeric antigen receptor T-cell therapy. Blood Adv. (2020) 4:2158–62. doi: 10.1182/bloodadvances.2020001450, PMID: 32428218 PMC7252550

[35] Herr PM Brantl V Priglinger SG Foerster P Thurau S . Severe visual impairment following CAR T-cell therapy in two individuals with Malignant optic nerve infiltration. Ocul Immunol Inflamm. (2024) 32:2280–4. doi: 10.1080/09273948.2024.2333393, PMID: 38579172

[36] Zhao X Liu Y Qin G Ge Y Li Q Chen X . Pseudoprogression in CAR-T cell therapy for solid tumor: Case report. Front Immunol. (2025) 15:1504104. doi: 10.3389/fimmu.2024.1504104, PMID: 39845944 PMC11750779

[37] Schroeder T Martens T Fransecky L Valerius T Schub N Pott C . Management of chimeric antigen receptor T (CAR-T) cell-associated toxicities. Intensive Care Med. (2024) 50:1459–69. doi: 10.1007/s00134-024-07576-4, PMID: 39172238 PMC11377606

[38] Ruff A Ballard HJ Pantel AR Namoglu EC Hughes ME Nasta SD . 18F-fluorodeoxyglucose positron emission tomography/computed tomography following chimeric antigen receptor T-cell therapy in large B-cell lymphoma. Mol Imaging Biol. (2021) 23:818–26. doi: 10.1007/s11307-021-01627-8, PMID: 34231105 PMC8578305

[39] Xia X Yang Z Lu Q Liu Z Wang L Du J . Reshaping the tumor immune microenvironment to improve CAR-T cell-based cancer immunotherapy. Mol Can. (2024) 23:175. doi: 10.1186/s12943-024-02079-8, PMID: 39187850 PMC11346058

[40] Kankeu Fonkoua LA Sirpilla O Sakemura R Siegler EL Kenderian SS . CAR T cell therapy and the tumor microenvironment: Current challenges and opportunities. Mol Ther Oncolytics. (2022) 25:69–77. doi: 10.1016/j.omto.2022.03.009, PMID: 35434273 PMC8980704

[41] Cox MJ Lucien F Sakemura R Boysen JC Kim Y Horvei P . Leukemic extracellular vesicles induce chimeric antigen receptor T cell dysfunction in chronic lymphocytic leukemia. Mol Ther. (2021) 29:1529–40. doi: 10.1016/j.ymthe.2020.12.033, PMID: 33388419 PMC8058445

[42] Scholler N Perbost R Locke FL Jain MD Turcan S Danan C . Tumor immune contexture is a determinant of anti-CD19 CAR T cell efficacy in large B cell lymphoma. Nat Med. (2022) 28:1872–82. doi: 10.1038/s41591-022-01916-x, PMID: 36038629 PMC9499856

[43] Mattie M Grosso M Auguste A Arnaud D Locke FL Neelapu SS . Pre- and post-treatment immune contexture correlates with long term response in large B cell lymphoma patients treated with axicabtagene ciloleucel (axi-cel). Blood. (2023) 142. doi: 10.1182/blood-2023-187574, PMID: 41761659

[44] Semba T Ishimoto T . Spatial analysis by current multiplexed imaging technologies for the molecular characterisation of cancer tissues. Br J Can. (2024) 131:1737–47. doi: 10.1038/s41416-024-02882-6, PMID: 39438630 PMC11589153

[45] Monick S Rosenthal A . Circulating tumor DNA as a complementary prognostic biomarker during CAR-T therapy in B-cell non-hodgkin lymphomas. Cancers (Basel). (2024) 16:1881. doi: 10.3390/cancers16101881, PMID: 38791959 PMC11120115

[46] Frank MJ Hossain NM Bukhari A Dean E Spiegel JY Claire GK . Monitoring of circulating tumor DNA improves early relapse detection after axicabtagene ciloleucel infusion in large B-cell lymphoma: results of a prospective multi-institutional trial. J Clin Oncol. (2021) 39:3034–43. doi: 10.1200/JCO.21.00377, PMID: 34133196 PMC10166351

[47] Zou H Liu W Wang X Wang Y Wang C Qiu C . Dynamic monitoring of circulating tumor DNA reveals outcomes and genomic alterations in patients with relapsed or refractory large B-cell lymphoma undergoing CAR T-cell therapy. J Immunother Can. (2024) 12:e008450. doi: 10.1136/jitc-2023-008450, PMID: 38443094 PMC11146396

[48] Dean EA Kimmel GJ Frank MJ Bukhari A Hossain NM Jain MD . Circulating tumor DNA adds specificity to PET after axicabtagene ciloleucel in large B-cell lymphoma. Blood Adv. (2023) 7:4608–18. doi: 10.1182/bloodadvances.2022009426, PMID: 37126659 PMC10448428

[49] Danylesko I Shouval R Shem-Tov N Yerushalmi R Jacoby E Besser MJ . Immune imitation of tumor progression after anti-CD19 chimeric antigen receptor T cells treatment in aggressive B-cell lymphoma. Bone Marrow Transplant. (2021) 56:1134–43. doi: 10.1038/s41409-020-01156-y, PMID: 33268830 PMC8113054

[50] Sanmamed MF Perez-Gracia JL Schalper KA Fusco JP Gonzalez A Rodriguez-Ruiz ME . Changes in serum interleukin-8 (IL-8) levels reflect and predict response to anti-PD-1 treatment in melanoma and non-small-cell lung cancer patients. Ann Oncol. (2017) 28:1988–95. doi: 10.1093/annonc/mdx190, PMID: 28595336 PMC5834104

